# Coronary flow reserve in stress-echo lab. From pathophysiologic toy to diagnostic tool

**DOI:** 10.1186/1476-7120-3-8

**Published:** 2005-03-25

**Authors:** Fausto Rigo

**Affiliations:** 1 Department of Cardiology Umberto I° Hospital Mestre-Venice, Italy

## Abstract

The assessment of coronary flow reserve by transthoracic echocardiography has recently been introduced into clinical practice with gratifying results for the diagnosis of left anterior descending artery disease simultaneously reported by several independent laboratories. This technological novelty is changing the practice of stress echo for 3 main reasons. First, adding coronary flow reserve to regional wall motion allows us to have – in the same sitting – high specificity (regional wall motion) and a high sensitivity (coronary flow reserve) diagnostic marker, with an obvious improvement in overall diagnostic accuracy. Second, the technicalities of coronary flow reserve shift the balance of stress choice in favour of vasodilators, which are a more robust hyperemic stress and are substantially easier to perform with dual imaging than dobutamine or exercise. Third, the coronary flow reserve adds a quantitative support to the exquisitely qualitative assessment of wall motion analysis, thereby facilitating the communication of stress echo results to the cardiological world outside the echo lab. The next challenges involve the need to expand the exploration of coronary flow reserve to the right and circumflex coronary artery and to prove the additional prognostic value – if any – of coronary flow reserve over regional wall motion analysis, which remains the cornerstone of clinically-driven diagnosis in the stress echo lab.

## Background and clinical perspectives

**Figure 1:**
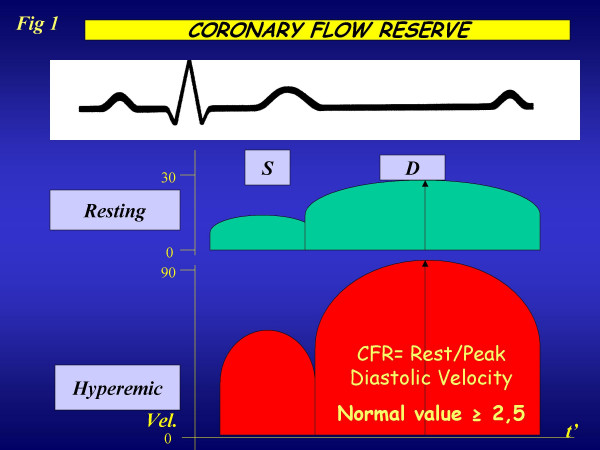
Schematic representation of coronary flow velocity profile obtained with tranthoracic Doppler of distal left anterior descending coronary artery : in diastole the flow velocity is higher than in systole.

**Figure 2:**
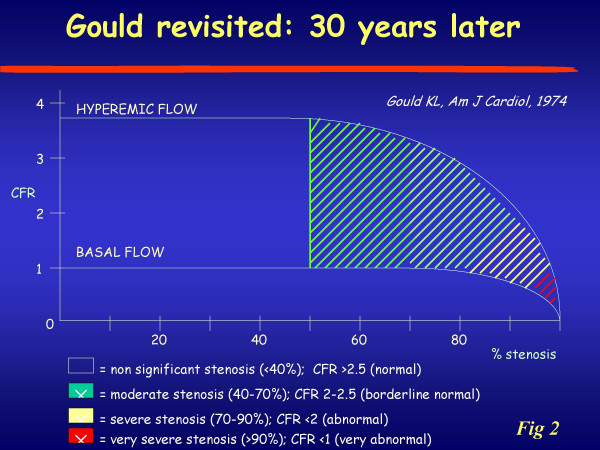
Relationship between the true increments of the flow signal obtained with the currently available imaging techniques. Modified from Gould KL, ref.1. On the abscissa are represented different narrowing of the coronary vessel.

Normally coronary blood flow can increase approximately four-to-six fold to meet increasing myocardial oxygen demands. This effect is mediated by vasodilation at the arteriolar bed, which reduces vascular resistance, thereby augmenting flow. The coronary reserve (CFR) represents the capacity of the coronary circulation to dilate following an increase in myocardial metabolic demands and can be expressed by the difference between the hyperemic flow and the resting flow curve (figure 1). In 1974, Lance K Gould [1] proposed the relationship between the anatomic condition and behaviour of coronary hyperaemic flow (figure2), whereby an inverse curvilinear relationship exists between the narrowing of lumen of coronary artery and hyperaemic capability, up to a completely abolished coronary reserve for stenosis >90%. This experimental paradigm can be accurately reproduced clinically in highly selected series of patients with single vessel disease, no myocardial infarction, no coronary collateral circulation, normal baseline function, no left ventricular hypertrophy, without evidence of coronary vasospasm, and off therapy at the time of testing. The perfect, predictable relationship found in the experimental animal or in a very selected patient population [2] is not so perfect in clinical practice [3,4], where many variables can modulate the imperfect match between epicardial coronary artery stenosis and coronary flow reserve (figure 2), such as the geometric characteristics of the stenosis, the presence of coronary collateral circulation, the microvascular component of coronary resistance, the presence of left ventricular hypertrophy modulating the myocardial extravascular component of coronary resistance, the viable or necrotic state of the myocardium distal to the stenosis, the presence of coronary macrovascular or microvascular spasm, and, last but not least, the presence of concomitant anti-ischemic therapy.

## Coronary flow reserve in the stress- echo lab: a new approach to CAD

**Figure 3:**
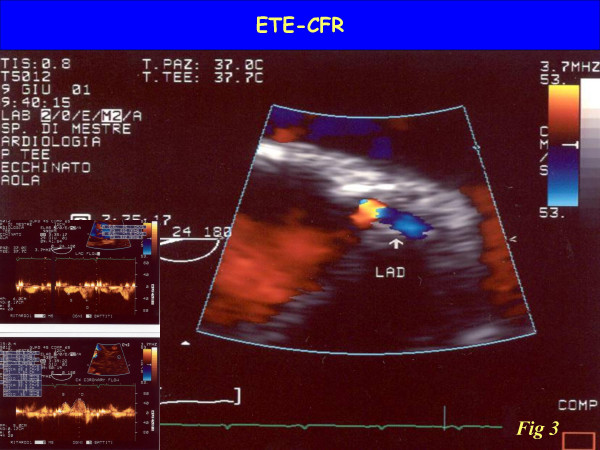
Visualization of left main and bifurcation of left anterior descending coronary artery and circumflex assessed by transesophageal approach. The color-Doppler trace the flow inside the proximal tract of left coronary artery Left: the Pulsed wave Doppler highlights the typical biphasic flow velocity coronary pattern

**Figure 4:**
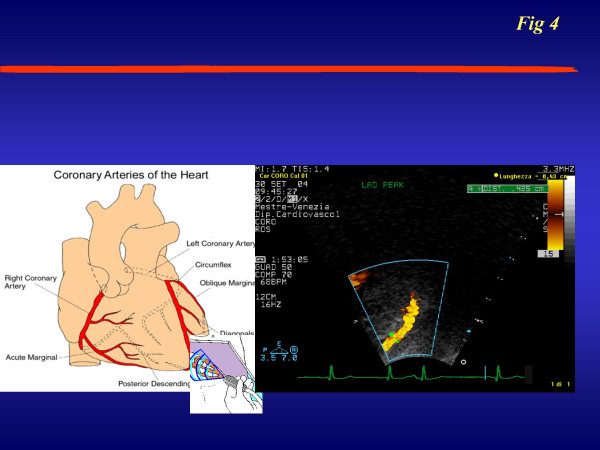
Artist's drawing illustrating transducer beam orientations to the left anterior descending coronary artery (LAD) with the corresponding echocardiographic images of the mid-distal tract of LAD color flow.

Up to now, coronary flow reserve has been evaluated invasively in the cath. lab and in nuclear medicine through perfusion imaging. Only recently has coronary flow reserve entered the echo lab, with the combination of coronary flow assessment by Doppler and vasodilator stress. With either TEE (sampling proximal tract) (figure 3) or TTE (exploring mid-distal tract) (figure 4), the coronary blood flow velocity profile recoded with pulsed wave Doppler is consistent with the pathophysiological premises. Accordingly, coronary flow velocity by Doppler assessment appears to be biphasic, with a lower peak during systole and a higher peak during diastole (figure 1). Myocardial extravascular resistance is higher in systole and lower in diastole due to the effect of myocardial contraction. The flow velocity variations are proportional to the total blood flow if the vessel lumen is kept constant, a reasonable assumption with the administration of drugs such as dipyridamole or adenosine. The coronary flow velocity variation between the baseline and peak effect of a coronary vasodilator allows a coronary flow reserve index in the left anterior descending artery territory to be derived. Peak diastolic flow is the simplest parameter to be measured and the most easily obtained, in addition to being the most reproducible and the one with the closest correlation with coronary perfusion reserve measured by positron emission tomography.

The coronary flow signal on LAD was first made possible by TEE (figure 3) – with excellent diagnostic results [5,6] – but only more recently has there been an increase in clinical interest due to the development of the transthoracic method [7-12]. There were technological factors which allowed the totally noninvasive transthoracic imaging of mid-distal LAD (figure 4): second harmonic imaging, allowing better definition of smaller structures, such as LAD; high frequency transducers (up to 8 MHz in second harmonic), leading to improved resolution imaging of near-field structures. The availability of contrast agents also improved the signal-to-noise ratio, thereby increasing the feasibility of transthoracic imaging of LAD above the threshold of potential clinical impact, although it is true that after a training period its use may not be necessary.

The Doppler assessment of coronary flow reserve has some limitations. The assessment of absolute blood velocity can be limited in some patients by the large incident angle between the Doppler beam and blood flow. However, calculation of the flow reserve allows assessment of flow patterns without the need for absolute values. More importantly, the velocity ratio is used as a surrogate of flow reserve: flow within the coronary artery is not calculated because cross-sectional visualization of the vessel does not allow an accurate measurement of the diameter of the vessel. The estimated flow reserve can be accurate if the coronary functions only as a conduit, without changing in diameter during drug infusion. This assumption is reasonable with dipyridamole [6] and less valid with dobutamine: this is an additional reason to stress coronary flow reserve with vasodilators.

## Coronary flow reserve: a new diagnostic power

The use of CFR as a "stand-alone" diagnostic criterion suffers from so many structural limitations as to make it little more than an academic somersault: firstly, only LAD is sampled; secondly, the coronary flow reserve cannot distinguish between microvascular and macrovascular coronary disease [13]. Therefore, it is much more interesting (and clinically realistic) to evaluate the additive value over conventional wall motion for LAD detection. The assessment of CFR adds sensitivity for LAD disease – with a modest loss in specificity. In reality, the inherently quantitative information of LAD flow reserve allows a stratification of the response, integrating many different tests into one: greatly reduced CFR (<1.5) yields extraordinary specificity whilst mildly reduced CFR (<2.0) offers outstanding sensitivity (Table 1).

**Table 1:** Diagnostic value of 2D Echo and coronary flow reserve

	**Sensitivity**	**CI 95%**	**Specificity**	**CI 95%**	**Accuracy**	**CI 95%**
**2D Echo**	74%	64–84%	91%	87–96%	86%	82–91%
**Coronary Flow Reserve (cut-off = 2)**	89%	81–96%	77%	71–84%	81%	76–86%
**Coronary Flow Reserve (cut-off = 1,9)**	81%	72–90%	84%	79–90%	83%	79–88%
**Coronary Flow Reserve (cut-off = 1,8)**	69%	58–79%	90%	85–95%	83%	79–88%
**Coronary Flow Reserve (cut-off = 1,7)**	63%	52–74%	97%	94–99%	86%	82–91%
**Coronary Flow Reserve (cut-off = 1,6)**	50%	38–61%	100%	-	85%	80–89%
**Coronary Flow Reserve (cut-off = 1,5)**	30%	19–41%	100%	-	79%	73–84%
**2D Echo / CFR cut-off = 1,9 **	90%	81–98%	94%	91–98%	93%	89–97%

**Figure 5:**
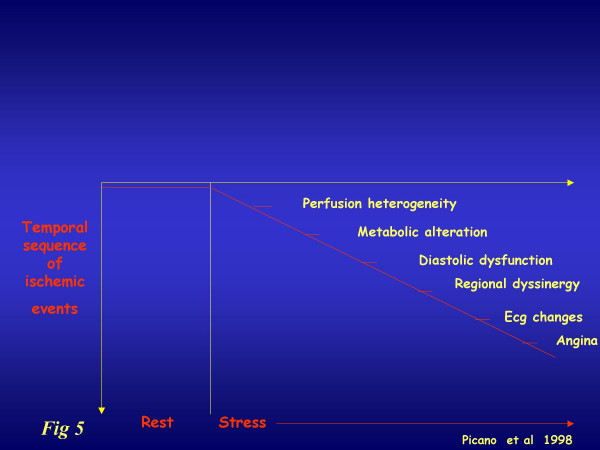
The classical ischemic cascade, triggered by coronary vasospasm and/or epicardial stenosis. The various markers are usually ranked according to a well-defined time sequence.

In addition, the flow information is relatively unaffected by concomitant antianginal therapy, which markedly reduces the sensitivity of ischemia-dependent regional wall motion abnormality [14] and does not influence coronary flow reserve, or does so only to a limited extent [15,16]. As a result, CFR can already help in the difficult task of identifying patients with coronary artery disease in accordance with the classic ischemic cascade (figure 5).

## Clinical application of coronary flow reserve

**Figure 6:**
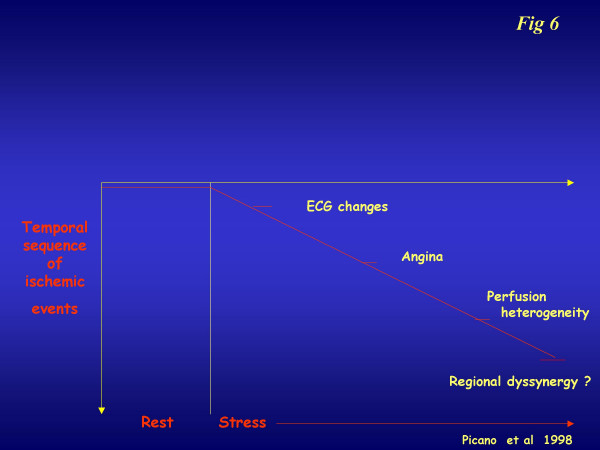
The alternative ischemic cascade, triggered by microvasculature dysfunction. The various markers are a different time sequence in comparison with the classical ischemic cascade.

In our experience of a single center since 2000, the combination of the CFR study on LAD and the analysis of left ventricular wall motion abnormalities represents the best choice in echo-lab evaluation of the flow-function relationship [17]. This can be done with the employment of a high frequency (5–7 MHz) probe aimed at investigating the coronary flow on LAD, and a low frequency (1,8–2,5 MHz) probe aimed at investigating the deeper wall motion behaviour. The presence of microvasculature dysfunction can create a bias between these two parameters: as a result, the WMSI guarantees good specificity while the CFR guarantees an improvement in sensitivity (Table 1). When the CFR cut-off value was decreased to below 2, the close relationship became even more evident (Table 1). Coronary flow reserve integrates and complements, but cannot be considered alternative to, classical stress echo based on regional wall motion analysis. Coronary flow reserve in the echo lab is not a "stand-alone" variable. In the echo lab, there is already stress echo information on wall motion: every additional piece of information, which adds to the overall complexity, should be evaluated in terms of its ability to provide additional information on wall motion analysis, which has high diagnostic accuracy and strong prognostic stratification power. Theoretically, and on the basis of the classic and alternative cascade (figure 6), coronary flow reserve information can be especially helpful for mild to moderate stenosis (capable of reducing flow reserve, but to subischemic levels) and in identifying patients with microvascular disease (reduced flow reserve and normal coronary arteries).

The combination of regional wall motion and coronary flow reserve identifies distinct patterns with 2 different parameters, coronary flow reserve and regional wall motion analysis. At one end of the spectrum, there is the totally normal pattern, with normal left ventricular function and normal coronary flow reserve, which is highly predictive of normal coronary anatomy and normal physiological response of coronary micro and macrocirculation. At the opposite end of the spectrum, there is abnormal left ventricular functional and abnormal coronary flow response, which is highly predictive of diseased epicardial coronary anatomy and impaired flow reserve. In between these extreme black-and-white responses, "gray zone" responses can be found, with mild-to-moderate abnormal coronary flow reserve and normal function with normal coronary flow reserve.

From PubMed search, we identified all papers with head-to-head comparison of dipyridamole stress echo (0.84 mg/kg ± atropine, with standard wall motion analysis) versus dipyridamole stress echo (0.84 mg/kg in 10' plus atropine or 0.84 mg/kg in 6' without atropine) with wall motion analysis and CFR evaluation. A total of 5 papers (from Italy, Argentina, and Japan) were found, involving 725 patients (355 with LAD stenosis and 370 without CAD [17-21](table 2).

**Table 2:** Meta-analysis on CFR and WMSI diagnostic value

	**SENSITIVITY (%)**		**SPECIFICITY (%)**		**ACCURACY (%)**	
	***DIP-2D***	***Dip-2D + CFR***	***DIP-2D***	***Dip-2D +CFR***	***DIP***	***Dip-2D + CFR***

Rigo et al, Am J Cardiol 2003	74	90	91	94	82	93
Lowenstein et al, JASE 2003	69	87	91	73	81	80
Nohtomi et al, JASE 2003	72	93	95	70	82	83
Chirillo et al, AJC 2004	67	93	91	93	71	93
Ascione et al, Int J cardiol 2004	51	83	96	98	78	94
	67 ± 9	90 ± 3	93 ± 2	86 ± 12	79 ± 5	89 ± 7

If we consider the latest papers on the diagnostic role of the Dipirydamole stress-test, it becomes clear that by adding the CFR-LAD evaluation to wall motion analysis we significantly increase the sensitivity of the test, whilst maintaining an excellent value in terms of specificity. As a result, diagnostic accuracy value is also extremely good. Up to now, this kind of relationship has only been valid for the LAD coronary artery, although this of course represents the most important coronary vessel in terms of clinical and prognostic impact.

## Coronary flow reserve: the importance of being quantitative

Coronary circulation has in itself the possibility of modulating blood flow variation in response to metabolic requests. The magnitude of this adaptive variation may be objectively expressed as a number such as coronary flow velocity reserve. The potential impact of this number still has to be clarified in the clinical arena.

Since 2000 we have evaluated the CFR-LAD behaviour of 1235 patients consecutively in our stress echo lab. We obtained the values summarized in Table 3, ranging from athletes to normal subjects and in the different patient subsets. It is important to underline the fact that different pathologies can give the same values in terms of CFR. Consequently, this functional parameter also has its own gray zone where it is often not possible to discriminate between dysfunction due to microvasculature or epicardial coronary disease. In our experience we found a significant impairment of CFR (< 2), in the three major cardiac pathologies with microvasculature dysfunction; in percentage terms, in 26% for Syndrome X, in 21% for Hypertrophic Cardiomyopathy and in 41% for Dilated Cardiomyopathy.

**Table 3:** Results on CFR in different pathologies

	Number	Male/Female	Mean age(years)	CFR
Normal patients	76	47/29	39 ± 12	3,32 ± 0,3
Syndrome X	97	24/73	57 ± 17	2,27 ± 0,3
LAD (≥ 70%)	223	171/152	63 ± 16	1,38 ± 0,2
LAD (<70%)	128	84/44	62 ± 16	2,2 ± 0,24
Hypertensive pts	323	72/251	56 ± 17	2,46 ± 0,44
DC	48	29/19	64 ± 112	1,94 ± 0,24
HCM	44	35/9	53 ± 11	2,21 ± 0,23
Aortic stenosis	22	6/14	74 ± 13	2,18 ± 0,34
Aortic insufficiency	12	5/7	68 ± 12	2,57 ± 0,40
PCI- LAD (>3 mo.)	72	51/21	61 ± 16	2,52 ± 0,45
Graft-IMA (>3 mo.)	56	41/15	64 ± 14	2,60 ± 0,38
Post-AMI (>3 mo.)	93	69/24	68 ± 17	1,98 ± 0,41
Athletes	41	41	34 ± 12	4,5 ± 0,45

LAD = Left anterior descending coronary artery; Syndrome X = microvasculature dysfunction; DC = Dilated cardiomyopathy; HCM = Hypertrophic cardiomyopathy; PCI-LAD = after LAD angioplasty (>3 months); Graft-IMA = By-pass with internal mammary artery on LAD; Post-Ami = after anterior myocardial infarction not revascularized.

## Prognostic role of coronary flow reserve

The additional diagnostic value of coronary flow reserve is represented by the possibility of monitoring different heart pathologies objectively and thereby obtaining important functional information over time in patient follow-up. Although the importance of the prognostic role played by stress-echo in patients with coronary artery disease has been demonstrated, there has also been emphasis on how the presence of antiischemic therapy at the time of testing can heavily modulate the predictive value of pharmacological stress-echo. In fact, a positive test in therapy is more prognostically malignant, and a negative test less prognostically benign [22]. The potential prognostic role of coronary flow reserve has recently been tested in predicting different clinical situations through invasive and nonivasive approaches. In particular, it has been demonstrated that the patency over time of coronary vessel disease after coronary angioplasty (PCI) can be accurately predicted by evaluating the functional status of the revascularized coronary artery just after the procedure [23-26]. Recently, we have emphasized the added prognostic role of an impairment of CFR despite normal wall motion contractility during combined dipyidamole stress-echo: these patients showed a worsening outcome during a mean follow-up of 24 months [27]. A further important application of CFR is as a good predictor of adverse events regarding the relationship with left ventricular remodelling after anterior myocardial infarction treated with coronary angioplasty [26-29]. In patients with coronary microvasculature dysfunction such as Dilated Cardiomyopathy (DC) [30-32] and Hypertrophic cardiomyopathy (HCM) [32,33], an impairment of CFR allows us to identify those patients with a worsening outcome and therefore represents an important guide to the efficient management of these patients.

Even if we need confirmation of the prognostic role of CFR through further and larger study, this parameter could have a very useful role to play in daily clinical practice.

## Multi coronary flow reserve: the next ultrasound challenge

At present, the impossibility of discriminating pathological behaviour of CFR due to microvascular or epicardial stenosis has represented a major limitation in the final diagnosis, as has the impossibility of investigating all the three major coronary arteries simultaneously [34].

**Figure 7:**
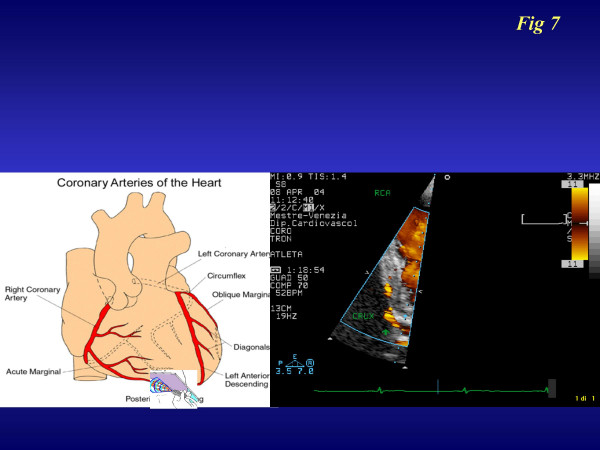
Imaging and projections for transthoracic imaging of right coronary and posterior descending coronary arteries.

We are trying to overcome this bias by introducing the contemporary analysis of at least two coronary arteries [35,36]. The most promising application regards right coronary evaluation, consisting of an apical off-axis 2 chamber approach: a high frequency probe (2^ Harmonic ;7 MHz receiving) [37] is rotated in a counter-clockwise position compared to the classical 3–4 chamber approach (figure 7). By applying this method in 658 consecutive patients (389 males; age 64,3 ± 13 years) referred for stress echocardiography, we were able to recognize a good right coronary flow (Additional file 1) and reserve in 429 pts (66%). We recognized the LAD-CFR (Additional file 2) in 637 pts (98%) from the same group: for Circumflex-CFR (Additional file 3) we were able to recognize a color flow signal in 344 pts (53%) but a good Pulse -Wave Doppler spectral in only 43% of patients. This last coronary artery creates the most difficulty in detecting a good pulse-Doppler signal during the peak test. This is due to the need to employ a low frequency probe which guarantees a better color Doppler signal but not such a good pulse Doppler signal due to wall noise interference.

## Coronary flow reserve evaluation in clinical practice

My 5-year personal experience of evaluating CFR added to wall motion abnormalities during a vasodilator stress-echo has led me to reach some final considerations. Such an integrated analysis is feasible and non time-consuming in any stress-echo Lab., after a short training period. By integrating these two pieces of information, we can improve our diagnostic and prognostic accuracy as together they offer a better and more complete pathophysiologic finding. This allows us to choose the best treatment for patients with coronary artery disease and to follow them up over time.

I believe that soon we will be in a position to make an accurate and exhaustive study of the posterior coronary artery, too and therefore to obtain a more complete functional cardiac evaluation. It follows that the integrated study of coronary flow and reserve and contractility will, in the near future, become the test of choice in guiding decision-making in the treatment of coronary stenosis before undergoing interventional procedures.

## List of Abbreviations

CFR-coronary flow reserve

LAD-left anterior descending artery

RCA-right coronary artery

WMSI-wall motion score index

## Supplementary Material

Additional File 1 Visualization by color-Doppler of the mid-distal tract of the right coronary artery and posterior descending with perforans branches. Click here for file

Additional File 2 Imaging of the blood flow inside the left anterior descending coronary artery depicted by color-Doppler in real anatomical direction. Click here for file

Additional File 3 Visualization by color-Doppler of the proximal-mid tract blood flow of Circumflex coronary artery in real anatomical direction. Click here for file
